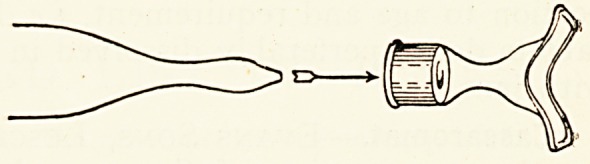# Notes on Preparations for the Sick

**Published:** 1912-03

**Authors:** 


### Notes on Preparations for the Sick.

**A New Nasal Attachment for " Vaporole " Ammonium Chloride Inhaler.**—BURROUGHS, WELLCOME & CO., London.—Ammonium Chloride vapour is usually inhaled by the mouth, but in some cases it is preferable to inhale through the nostrils and allow the vapour to escape by the mouth. For this purpose a new nasal attachment has been introduced. Instead of twin bulbs to go inside the nares an expanded orifice with flanged edges is used. It is made of glass, and so shaped as to be adapted to the physical configuration of any patient.

The orifice is placed in position beneath the nose, and the depression in the centre permits the flanged edges to enclose the outer edges of the nares.

This method is to be preferred both on æsthetic and hygienic grounds. The new nasal attachment is fitted with a rubber plug for the reception of the ordinary mouth-piece of the "Vaporole" Ammonium Chloride Inhaler, thus rendering the complete outfit suitable for either nasal or oral inhalations. A noteworthy feature of the "Vaporole" inhaler is the exact measurement of ammonia and acid which is effected by supplying the requisite quantity of each in atomic proportions in two hermetically-sealed glass containers covered with gauze. These "Vaporole" containers are fractured and placed in a tube, and the mingled vapours, automatically filtered and supplied with sufficient moisture, are inhaled in a pure and neutral condition.

**Colloid Metal Therapy: "Collosol" Argentum, "Collosol" Hydrargyrum** (Crooke's).—OPPENHEIMER, SON & CO., London.—These solutions of metals in a colloid state have very remarkable therapeutic properties, the actual particles of metal present are in an extremely minute state, they cannot be removed from the liquid by filtration as they pass readily through the pores of the filter, by ultramicroscopic examination (they are invisible under the ordinary microscope) these particles are seen to be remarkably even in size, and they exhibit marked Brownian movement. Physical tests show that these metals are included under the classification of Reversible Colloids. Perhaps the most remarkable property of "Cellosols" is their absolute freedom from toxic effects to the human organism, notwithstanding their very high bactericidal power

These special Colloid solutions of the metals are manufactured solely in the Crooke's Laboratory. The most striking advantage they possess over ordinary Colloid solutions is their stability; they are not precipitated by the blood. The sphere of utility of these preparations is practically unlimited. Though of recent introduction, they have already proved to be the treatment *par excellence* for the many diseases in which their use is specially indicated. They are used by hypodermic injection or by the mouth, and it is stated that they are powerfully antiseptic and bactericidal.

**Agarase:** a combination of Agar-Agar with Bulgarian Lactic Ferment.—ROBERTS & Co., New Bond Street, London.—It is prepared in tablet form and coated with Keratine. The average dose is two to three tablets with each of the three principal meals. They are said to be an efficient intestinal regulator, giving a rational treatment of constipation with no violent effects.

**Peristaltin "Ciba":** an aperient glucoside of Cascara-Sagrada Bark, soluble in water.—SOCIETY OF CHEMICAL INDUSTRY IN BASLE.—Adults take one tablet two or three times daily, or two to three tablets at one time in the afternoon or evening.

For continual use, the desired result can be attained with two tablets daily, often only with one.

Children[1] are given a correspondingly smaller dose in proportion to age and requirement, *i.e.* half to one, or one to two tablets daily, perferably dissolved in sugar water or mixed up with jam, etc.

**Cascaromat.**—EVANS SONS, LESCHER & WEBB, London.—Another preparation of Cascara which is very effective and useful.

**Thaolayine.**—ROBERTS & Co., London.—Another laxative of the Cascara type, not an immediate laxative or purgative, but a regular result is commonly obtained after several days' use. It is prepared as scales, cachets, compressed tablets and granules.

**Ovogal.**—J. D. RIEDEL Co., London, E.C.—A new cholagogue for biliary disorders. Much clinical evidence is given in favour of this albuminate compound with bile acids. It is strongly recommended for cases of cholelithiasis and other intestinal disorders. It is supplied in capsules containing half a gramme. One to three may be given several times daily.

**Sal Antisepticus.** (Huxley).—ANGLO-AMERICAN PHARMACEUTICAL Co., Croydon.—An alkaline antiseptic powder. A ready means of quickly preparing lotions, gargles and injections.

In 5 per cent. solution is equal to 1 in 1,000 solution of bichloride of mercury as a germicide, and superior to 2 per cent. solution of carbolic acid. The mild but bland astringency of this combination of antiseptics ensures freedom from locking up septic matter in the tissues.

It supplies a definite combination of reliable antiseptics in a dry granular condition ready for diluting as wanted, and is free from the dangers of corrosive carbolic acid of bichloride of mercury.

[1] Peristaltin chocolate is especially recommended for children on account of its pleasant form of administration.

## Figures and Tables

**Figure f1:**
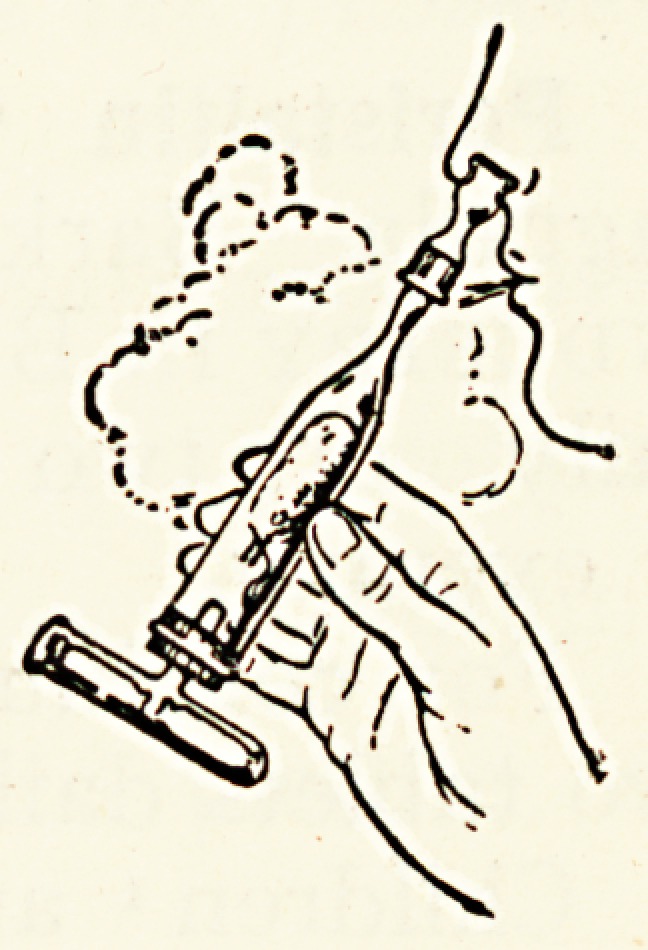


**Figure f2:**